# Interaction between hydrogen sulfide, nitric oxide, and carbon monoxide pathways in the bovine isolated retina

**DOI:** 10.3934/Neuroscience.2019.3.104

**Published:** 2019-06-18

**Authors:** Madhura Kulkarni-Chitnis, Leah Mitchell-Bush, Remmington Belford, Jenaye Robinson, Catherine A. Opere, Sunny E. Ohia, Ya Fatou N. Mbye

**Affiliations:** 1 Department of Pharmaceutical Sciences, College of Pharmacy and Health Sciences, Texas Southern University, Houston, TX 77004, USA; 2 Department of Pharmacy Sciences, School of Pharmacy and Health Professions, Creighton University, Omaha, NE 68178, USA

**Keywords:** gasotransmitters, retina, endogenous production, hydrogen sulfide, carbon monoxide, nitric oxide

## Abstract

**Purpose:**

Nitric oxide (NO), carbon monoxide (CO) and hydrogen sulfide (H_2_S) are physiologically relevant gaseous neurotransmitters that are endogenously produced in mammalian tissues. In the present study, we investigated the possibility that NO and CO can regulate the endogenous levels of H_2_S in bovine isolated neural retina.

**Methods:**

Isolated bovine neural retina were homogenized and tissue homogenates were treated with a NO synthase inhibitor, NO donor, heme oxygenase-1 inhibitor, and/donor. H_2_S concentrations in bovine retinal homogenates were measured using a well-established colorimetric assay.

**Results:**

L-NAME (300 nM–500 µM) caused a concentration-dependent decrease in basal endogenous levels of H_2_S by 86.2%. On the other hand, SNP (10–300 µM) elicited a concentration-related increase in H_2_S levels from 18.3 nM/mg of protein to 65.7 nM/mg of protein. ZnPP-IX (300 nM–10 µM) caused a concentration-dependent increase in the endogenous production of H_2_S whereas hemin (300 nM–20 µM) attenuated the basal levels of H_2_S.

**Conclusion:**

We conclude that changes in the biosynthesis and availability of both NO and CO can interfere with the pathway/s involved in the production of H_2_S in the retina. The demonstrated ability of NO, CO and H_2_S to interact in the mammalian retina affirms a physiological/pharmacological role for these gaseous mediators in the eye.

## 1. Introduction

The presence of enzymes responsible for the biosynthesis of hydrogen sulfide (H_2_S) from the amino acid, L-cysteine in mammalian tissues has stimulated a surge of interest in its potential physiological significance in biological processes [1],[2]. There is evidence that H_2_S can share similar physiological and pharmacological actions with other gaseous transmitters such as nitric oxide (NO) and carbon monoxide (CO) [3]–[5]. In some pathophysiological conditions, the three gaseous molecules are present in specific concentrations in disease states such as Alzheimer's when their endogenous production is known to be compromised [6]–[8]. Although these gases possess similar features and modes of action, H_2_S has been reported to exhibit a number of distinct characteristics from both NO and CO [7]. For instance, H_2_S has greater water solubility and the lowest potential to penetrate lipid bilayers, and has a different pathway that leads to its biosynthesis when compared with its two gaseous counterparts [9]. The basal production of H_2_S is dependent upon the activity of two pyridoxal-5′-phosphate dependent-enzymes, cystathionine β-synthase (CBS) and cystathionine γ-lyase (CSE), whilst NO is synthesized from L-arginine by NO synthase and CO is endogenously produced from heme by heme oxygenase [10]–[15]. In the presence of aspartate aminotransferase, L-cysteine can also be converted to H_2_S by the enzyme, 3-mercaptopyruvate sulfur-transferase (3MST) [16]–[20]. Since the physiological and pharmacological targets for H_2_S are analogous to NO and CO, evidence available from literature supports an interaction between these gaseous transmitters in mammalian organs and tissues [7],[21],[22]. Indeed, studies in neuronal tissues and the vasculature show that the interaction between these gases can affect their biosynthetic pathways, as well as, induce changes in their biological responses [23]. While H_2_S has been shown to alter the activity and expression of NO producing enzyme (eNOS), there is evidence that NO substrate or donors can also modulate expression and activity of H_2_S producing enzymes [3],[7],[24]–[26]. Furthermore, studies by Whiteman et al. [27] suggest that H_2_S may interact with NO to form an unidentified nitrosothiol moiety, which may then regulate physiological functions of both NO and H_2_S [24]. Likewise, there is compelling evidence of an interaction between H_2_S and CO. H_2_S has been reported to play a regulatory role in CO/heme oxygenase pathways in cardiac and pulmonary diseases [28]. Indeed, under physiological conditions, CO/heme oxygenase and H_2_S/CSE pathways have been demonstrated to inhibit each other in aortic smooth muscle cells [29]. However, it remains to be determined whether NO and CO can interact with the pathway leading to H_2_S biosynthesis in the retina. The aim of the present study was, therefore, to investigate whether inhibitors and/or activators of both NO and CO pathways can alter H_2_S concentrations in the bovine isolated neural retina. Parts of the work described in this paper have been communicated in an abstract form [30].

## 2. Materials and methods

### 2.1. Chemicals

Hemin, zinc protoporphyrin (ZnPP-IX), L-nitroarginine methyl ester (L-NAME), sodium nitroprusside (SNP), aminooxyacetic acid (AOAA), ethylenediamine tetra-acetic acid solution (EDTA), zinc acetate, ferric chloride, borate buffer (pH 11.0) and *N*, *N*-dimethyl-*p*-phenylenediamine sulfate were all purchased from Sigma–Aldrich, St. Louis, MO. All reagents were freshly prepared in distilled water, except *N*, *N*-dimethyl-*p*-phenylenediamine which was prepared in 7.2 M hydrochloric acid, immediately before use in the series of experiments.

### 2.2. Tissue preparation

Bovine eyes were obtained from Vision Tech, Dallas, TX within 24 hours following enucleation and transported to the laboratory on ice. An incision was made along the equator of each eye and the vitreous humor and lens were delicately removed. The neural retinae were isolated by gentle removal from the posterior segment of the eye and immediately immersed in ice-cold 50 mM potassium phosphate buffer (pH 7.4). Time elapsed between animal sacrifice and tissue preparation was less than 24 hours.

### 2.3. Measurement of basal hydrogen sulfide production in retinal tissues

H_2_S production was measured using the Methylene Blue method, a widely used assay that has been shown to produce quantifiable measurements of H_2_S production in mammalian tissues comparable to data obtained using H_2_S probes [31]–[35]. The methodology employed for H_2_S measurement in retinal tissues was essentially the same as previously reported by Xia et al. [36] and Kulkarni et al. [37], with some minor modifications. In brief, isolated bovine neural retina were homogenized in freshly prepared 50 mM ice-cold potassium phosphate buffer (pH 7.4) using a hand held tissue grinder consisting of a pestle and mortar. The tissue homogenates (0.25 ml) were incubated in the presence or absence of NO synthase inhibitor, L-nitroarginine methyl ester (L-NAME; 300 nM–500 µM), NO donor, sodium nitroprusside (SNP; 10–300 µM), hemoxygenase-1 inhibitor, zinc protoporphyrin (ZnPP-IX; 300 nM–10 µM) and hemoxygenase-1 inducer, hemin (300 nM–20 µM). The drug concentrations chosen were based on previously published reports on the pharmacological effects of NO and CO on mammalian tissues and cells [29],[37]. Incubation was carried at 37 °C for 90 min in tightly sealed eppendorf tubes for the enzymatic reaction to take place for optimum production of H_2_S as reported by [36],[37]. EDTA (0.125 ml; 5 mM) was injected into the reaction tubes to stop the reaction, followed by 0.5 ml of zinc acetate (1%) and 0.5 ml of borate buffer (pH 11.0). The tubes were sealed and then incubated at 37°C for another 30 minutes. The reaction solution was mixed with 0.5 ml of N, N-dimethyl-p-phenylenediamine sulfate (20 mM, in 7.2 M HCl) and 0.5 ml of FeCl3 (30 mM) at 37 °C for an additional 10 minutes and then centrifuged at 5000g for 3 min. The absorbance of resulting solution was measured at 670 nm with a spectrophotometer. The H_2_S concentration was calculated against the calibration curve of standard H_2_S solutions (10–750 µM). The protein content of the residual pellet after centrifugation was measured using the Bradford method.

### 2.4. Data analysis

Results were expressed as nanomolar of H_2_S produced per milligram soluble protein. Values given are arithmetic means ± SEM. The means were determined from three separate experiments performed in triplicates. Significance of differences between control and treatment groups were evaluated using one-way analysis of variance (ANOVA) followed by Newman-Keul comparison test. (Graph Pad Prism Software, San Diego, CA). The level of significance was chosen as *p* < 0.05.

## 3. Results

### 3.1. Effects of L-NAME and SNP on basal concentrations of H_2_S

**Figure 1.**
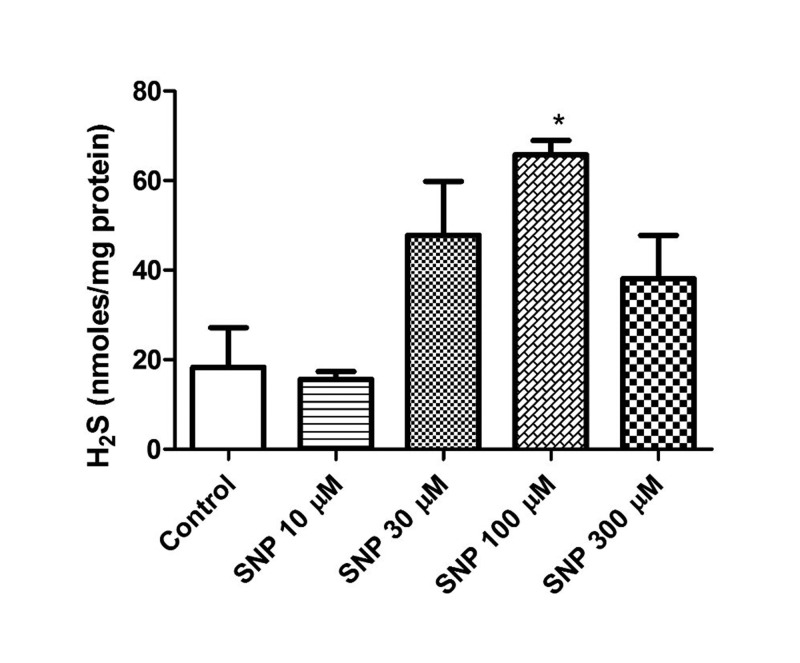
Effect of sodium nitroprusside (SNP) on endogenous levels of hydrogen sulfide in isolated bovine retina. Vertical bars represent means ± S.E.M; n = 9. *p < 0.05 and **p < 0.01, significantly different from control.

**Figure 2.**
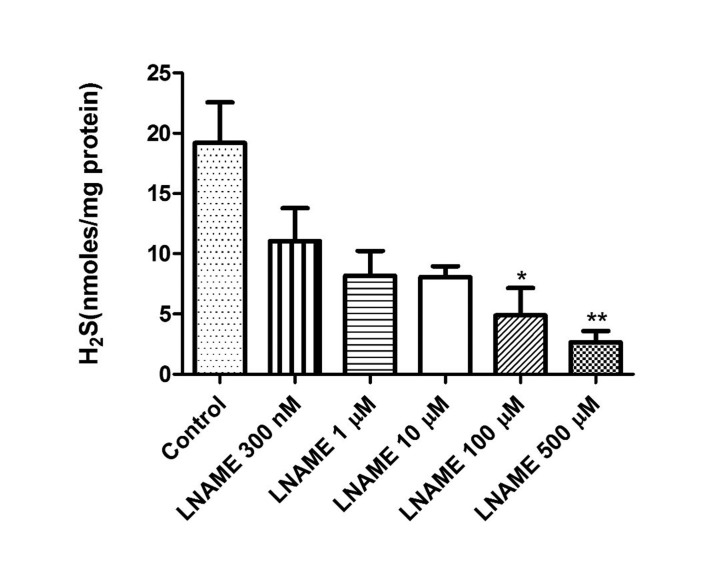
Effect of L-nitroarginine methyl ester (L-NAME) on endogenous levels of hydrogen sulfide in isolated bovine retina. Vertical bars represent means ± S.E.M; n = 9. *p < 0.05 and **p < 0.01, significantly different from control.

In a previous study, we showed evidence that endogenous concentrations of H_2_S in isolated bovine retinae were enhanced by the substrate, L-cysteine and attenuated by inhibitors of enzymes responsible for the biosynthesis of this gas [37]. In the present study, the NO donor, SNP (10–100 µM) caused a concentration-dependent increase in H_2_S levels reaching a maximum at 100 µM (65.7 nm/mg of protein) (Figure 1). On other hand, the NO inhibitor L-NAME (300 nM–500 µM) elicited a concentration-dependent decrease in the basal endogenous levels of H_2_S from 19.2 to 2.6 nm/mg of protein (Figure 2).

### 3.2. Effects of Hemin and ZnPP-IX on basal concentrations of H_2_S

**Figure 3.**
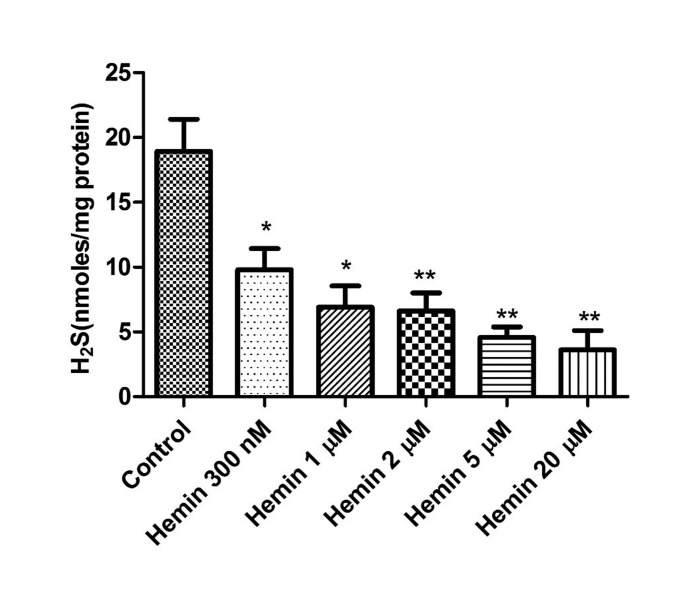
Effect of hemin on endogenous levels of hydrogen sulfide in isolated bovine retina. Vertical bars represent means ± S.E.M; n = 6. *p < 0.05, **p < 0.01 and ***p < 0.001, significantly different from control.

**Figure 4.**
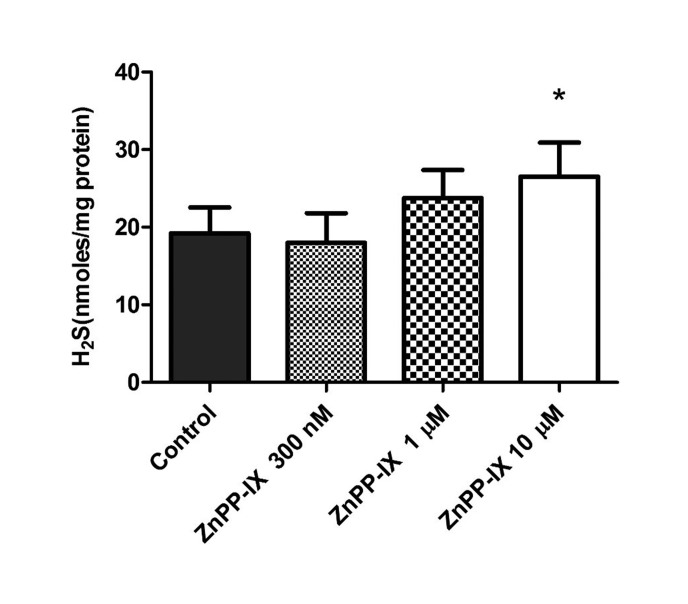
Effect of zinc protoporphyrin (ZnPP-IX) on endogenous levels of hydrogen sulfide in isolated bovine retina. Vertical bars represent means ± S.E.M; n = 6. *p < 0.05, **p < 0.01 and ***p < 0.001, significantly different from control.

We next investigated the pharmacological action of the heme oxygenase-1 inducer, hemin (300 nM–20 µM) on basal concentrations of H_2_S in the bovine isolated retina. As shown in Figure 3, hemin produced a concentration-dependent attenuation of basal levels of H_2_S from 18.9 to 3.6 nm/mg of protein. Conversely, the heme oxygenase inhibitor, ZnPP-IX (300 nM–10 µM) significantly increased the concentration of H_2_S from 19.2 to 26.5 nm/mg of protein in a concentration-dependent manner, reaching a maximal effect at 10 µM (Figure 4).

## 4. Discussion

It is well-established that H_2_S can serve as a gaseous neurotransmitter/neuromodulator in several mammalian tissues and organs. Indeed, there is abundant evidence that H_2_S is involved in several physiological and pathophysiological processes as diverse as learning and memory, inflammation, and the regulation of blood pressure [6],[38],[39]. In the cardiovascular system, H_2_S has been shown to play an important role in maintenance of vascular smooth muscle tone whereas in the central nervous system, this gas has been found to act as a neurotransmitter/neuromodulator at synapses [40]–[43]. Furthermore, H_2_S has also been reported to exert a neuroprotective action on neurons [11]. The observed biological actions of H_2_S in the vasculature and brain have been reported to resemble those of other established gaseous transmitters like CO and NO [44],[45]. Although, studies reported in literature have focused on understanding the physiological and pharmacological roles of H_2_S in cardiovascular, central nervous and immune systems, there is limited evidence of an interaction between NO, CO and H_2_S in regulating some of the observed responses in the above-named systems [8]. In the eye, changes in cyclic GMP levels have been reported due to an interaction between these gaseous transmitters in the salamander retina and the rabbit ophthalmic artery [46],[47]. In the present study, we investigated the possibility that activation or inhibition of pathways involved in NO and CO biosynthesis can alter basal endogenous levels of H_2_S in the bovine isolated retina.

There is evidence that in the cardiovascular and central nervous systems, nitric oxide synthase (NOS) substrates or NO donors can up-regulate the expression or activity of H_2_S producing enzymes [48]–[51]. Other investigators also report that L-arginine can attenuate pulmonary artery pressure by augmenting the expression and activity of CSE mRNA in lung tissue [52]. Furthermore, there is evidence suggesting that a synergistic interaction exists between NO and H_2_S in regulating vascular function [53]–[56]. In the present study, we measured endogenous levels of H_2_S in the presence of NO donor, SNP. The incubation time period for optimal production of endogenous basal levels of H_2_S was previously established in our laboratory [37]. We found that SNP increased the basal endogenous levels of H_2_S suggesting that increased NO availability can regulate the production of this gas in the retina. We also observed that at a high concentration of SNP (300 µM) the basal endogenous levels of H_2_S was decreased, suggesting that NO may also have the ability to inhibit H_2_S production. The precise mechanism for this effect is not clear. However, it may be due to the fact that at high concentrations of SNP, the NO generated binds to CBS and thus has the potential to inhibit its activity and therefore the generation of H_2_S. Despite this, our observations correlate well with studies in the vasculature depicting the ability of SNP to increase the biosynthesis of H_2_S [48]–[50]. Indeed, there is evidence that in the presence of NO, the pharmacological actions of H_2_S are significantly enhanced [8],[56]. The ability of NO to stimulate H_2_S production may be due to an augmented activity of enzymes responsible for the biosynthesis of H_2_S by the NO-induced S-nitrosylation of their cysteine residues or indirect stimulation of CSE by NO through an increase in the activity of cyclic GMP-dependent kinases [8],[35],[45]. Taken together, these results indicate that NO may interact directly with CBS and CSE to augment H_2_S production. To confirm the nature of this interaction, we investigated the effect of an inhibitor of NO synthase, L-NAME on endogenous H_2_S production. Our results demonstrate that L-NAME reduced basal H_2_S levels suggesting that endogenously generated NO can modulate the basal levels of H_2_S presumably through an interaction with pathways involved in H_2_S production. Other studies in the vasculature have also reported a similar inhibitory effect of L-NAME on H_2_S production and activity [8],[53]. The exact mechanisms +that underlie H_2_S and NO interactions in the neural retinae are unknown and merits further investigation.

In addition to studying the possible interaction between NO and H_2_S, we designed experiments to determine whether changes in CO production can regulate the endogenous levels of H_2_S in neural retinae. In these studies, hemin and ZnPP-IX were employed as an inducer and an inhibitor of heme oxygenase, respectively [57],[58]. We found that hemin caused a concentration-related decrease in basal levels of H_2_S. In contrast, ZnPP-IX elicited a concentration-dependent increase in endogenous H_2_S levels. Our results reveal that increasing CO production led to a decrease in basal concentrations of H_2_S whereas, inhibiting CO biosynthesis can cause an elevation of H_2_S levels. These results correlate well with studies reported by Jin et al. [29] who showed that endogenous CO down-regulates the H_2_S production in aortic smooth muscle cells. A similar interaction between these two gaseous transmitters has also been observed in the carotid body, the liver, and the microvasculature of the brain [59]–[61]. It appears that in the bovine retina, reduction of CO biosynthesis removes a tonic inhibitory action of this gas on H_2_S production. It is, however, unclear how CO acts to regulate the biosynthesis of H_2_S in the retina.

Previous evidence from our laboratory and others suggests that crosstalk between the three gasotransmitters exists in ocular tissues [46],[47],[62]–[64]. In the salamander retina, all three gases were found to play interactive roles in modulating retinal cGMP signaling, as NO and CO significantly increased cGMP-like immunoreactivity (cGMP-LI) both alone and synergistically [46]. NaHS was found to inhibit the NO-induced increase in cGMP-LI from the inner nuclear layer to the ganglion cell layer, indicating an interaction in these three gases in the retina [46]. H_2_S was found to enhance phenylephrine induced tone in the rabbit ophthalmic artery, while CO and NO both caused concentration dependent relaxations [47]. The mechanisms behind these pharmacological actions may be attributed to the gaseous transmitters' ability to modulate cGMP production in the rabbit model, as H_2_S was found to reduce cGMP levels and prevent a NO induced increase in cGMP [47]. The relaxing effect of hydrogen sulfide donors on smooth muscles in both bovine and porcine ocular tissues have also been found to be dependent, at least in part, on the presence and activity of nitric oxide [62]–[64]. Taken together, these studies implicate an interactive regulatory role of these gaseous modulators in ocular tissues.

In conclusion, changes in the biosynthesis and availability of both NO and CO can interfere with the pathway/s involved in the production of H_2_S in the bovine isolated retina. The present observation that basal levels of H_2_S can be altered by manipulation of pathways involved in NO/CO production is of great interest and merits further investigation.

Despite these novel findings, the results should be interpreted with prudence due to limitation of our study protocol. For instance the colorimetric methodology used for determination of endogenous production of H_2_S is based on the assumption that tissue concentration of sulfide are high, and hence it does not distinguish between acid labile sulfur stores or bound sulfane stores. In addition the enzyme inhibitors being used in the studies are pyridoxal-5′ phosphate (PLP) enzyme inhibitors that are not specific for H_2_S biosynthesizing enzymes, CBS and CSE as these enzymes are known to be involved in other cellular processes in mammals. Thus, it would be worthwhile to investigate whether H_2_S synthesis alters the expression of these biosynthesizing enzymatic proteins. Despite the limitations of the study protocol, the results provide a basis for future studies on the role of H_2_S in the posterior segment of the eye.
